# NSD1 governs H3K36me2-mediated DNA methylation and drives endo-mesodermal differentiation of human iPSCs

**DOI:** 10.1186/s13148-026-02162-5

**Published:** 2026-05-20

**Authors:** Anna Hansknecht, Martina Mankarious, Monica Varona Baranda, Esra Dursun Torlak, Wolfgang Wagner, Kira Zeevaert, Deepika Puri

**Affiliations:** 1 Helmholtz-Institute for Biomedical Engineering, Medical Faculty of RWTH Aachen University, 52074 Aachen, Germany; 2 Institute of Stem Cell Biology, University Hospital of RWTH Aachen, 52074 Aachen, Germany

**Keywords:** NSD1, H3K36me2, iPSCs, DNA methylation, Endodermal differentiation

## Abstract

Epigenetic regulatory mechanisms, which include histone modifications and DNA methylation, play a central role in development and aging. Dimethylation of H3K36, deposited mainly by the histone methyltransferase NSD1, occurs predominantly in intergenic regions, and recruits the DNA methyltransferase DNMT3A to facilitate DNA methylation. Haploinsufficiency of NSD1 results in the overgrowth disorder Sotos syndrome in vivo, which is associated with aberrant DNA methylation signatures and an enhanced epigenetic age. To understand the mechanisms by which NSD1 may regulate development, differentiation, and diseases, we generated human iPSC lines deficient in functional NSD1 (NSD1-KO). NSD1-KO cells exhibit a substrate-specific decrease in proliferation, reduced H3K36me2 levels, and extensive DNA hypomethylation. Notably, the loss of functional NSD1 altered the differentiation potential of iPSCs, with aberrant endodermal and mesodermal lineage commitment. Further analysis revealed that NSD1 might drive endodermal differentiation by regulating the expression of an endodermal lncRNA ‘HIDEN’ by mechanisms independent of the regulation of DNA methylation. Our NSD1-KO iPSC lines partially recapitulate the DNA methylation defects associated with NSD1-related developmental disorders. Additionally, we uncovered a novel mechanism by which NSD1 may regulate endodermal differentiation of human iPSCs.

**Supplementary Information:**

The online version contains supplementary material available at 10.1186/s13148-026-02162-5.

## Introduction

Epigenetic modifications are crucial in determining the activity and accessibility of genes and regulatory elements without altering the underlying DNA sequence [1]. Interactions between multiple epigenetic features, such as DNA methylation (DNAm) and histone modifications, play a central role during cellular development and maturation [2–4], and aberrations in these pathways contribute to the pathogenesis of various diseases, including cancer [5–7]. Therefore, understanding the epigenetic network is critical for elucidating the mechanisms governing differentiation, aging, and the development of pathogenic transcriptional activity.

In mammals, CG dinucleotides (CpG sites) are methylated in a regulated manner during cellular development and aging [8–11]. Recent studies have implicated histone modifications such as histone H3 lysine 36 (H3K36) methylation in the recruitment of DNA methyltransferase (DNMT) proteins and consequently the establishment of DNA methylation [12]. Dimethylation of H3K36 (H3K36me2) predominantly colocalizes with DNMT3A, especially in intergenic regions, playing a key role in the establishment of the DNA methylation pattern [12, 13]. H3K36me2 is deposited by several histone methyltransferases (HMTs), however, the Nuclear Receptor Binding SET Domain Protein 1 (NSD1) is one of the major enzymes establishing this histone mark in humans [12]. Underlining the role of NSD1 for early differentiation processes, homozygous NSD1-deficient mouse embryos display an impaired mesendoderm formation and fail to complete gastrulation [14]. Furthermore, studies in mouse embryonic stem cells implicate NSD1 function in stem cell differentiation processes [15, 16]. In humans, NSD1 dysfunction is linked to diseases such as Sotos syndrome, in which over 90% of the patients have abnormalities in NSD1 [17, 18]. Sotos syndrome is a childhood overgrowth syndrome characterized by distinctive facial features, physical overgrowth, advanced bone age, and learning disabilities [19, 20]. Genome-wide DNA methylation analysis in Sotos syndrome patients revealed a specific signature that distinguishes pathological NSD1 mutations from benign or control patterns, global DNA hypomethylation, especially in intergenic regions, and an acceleration of epigenetic age [12, 21, 22].

NSD1-mediated H3K36me2 has been reported to restrict polycomb repressive complex 2 (PRC2) activity and prevent aberrant polycomb-mediated gene silencing and early differentiation of stem cells through uncontrolled deposition of H3K27me3 [23]. Furthermore, reports indicate that NSD1 can bind to methylated H3K4 and H3K9, potentially impacting gene expression [24]. NSD1 also binds to enhancers and modulates the H3K27Ac levels, hence regulating enhancer activity [15, 16, 25]. These reports and more indicate that NSD1 is part of a complex interplay of epigenetic regulation that may play critical roles in gene expression and cellular function.

To better understand the interplay between NSD1-mediated H3K36me2 and DNA methylation and its impact on development and differentiation, we modified NSD1 in human induced pluripotent stem cells (iPSCs) using CRISPR/Cas9 gene editing and systematically reduced H3K36me2. Human iPSCs have the capacity for cellular differentiation into the three germ layers and all derived cell lines, such as mesenchymal stromal cells, which make them a valuable tool for disease modeling, analysis of early development and senescence-associated epigenetic changes [26]. Furthermore, DNA methylation profiles differ between naïve and primed pluripotent stem cells [27, 28], altering their potency, which warrants a better understanding of the role of NSD1-mediated DNA methylation in iPSC pluripotency. NSD1-modified cell lines retained pluripotency markers, whereas differentiation potential towards endodermal and mesodermal lineages was impaired. We found a significant downregulation of the Human IMP1-Associated “Desert” Definitive Endoderm lncRNA (*HIDEN*), which plays a significant role in endodermal differentiation of human pluripotent cells mediated by the WNT signaling pathway [29]. Furthermore, extensive DNA hypomethylation at intergenic regions showed overlapping patterns with Sotos syndrome patients. Overall, our results provide valuable insights into NSD1-mediated H3K36 dimethylation as a regulator for DNA methylation and differentiation potential of human iPSCs.

## Results

### Genetic modification of *NSD1* induces loss of H3K36 dimethylation in human iPSCs

**Fig. 1:**
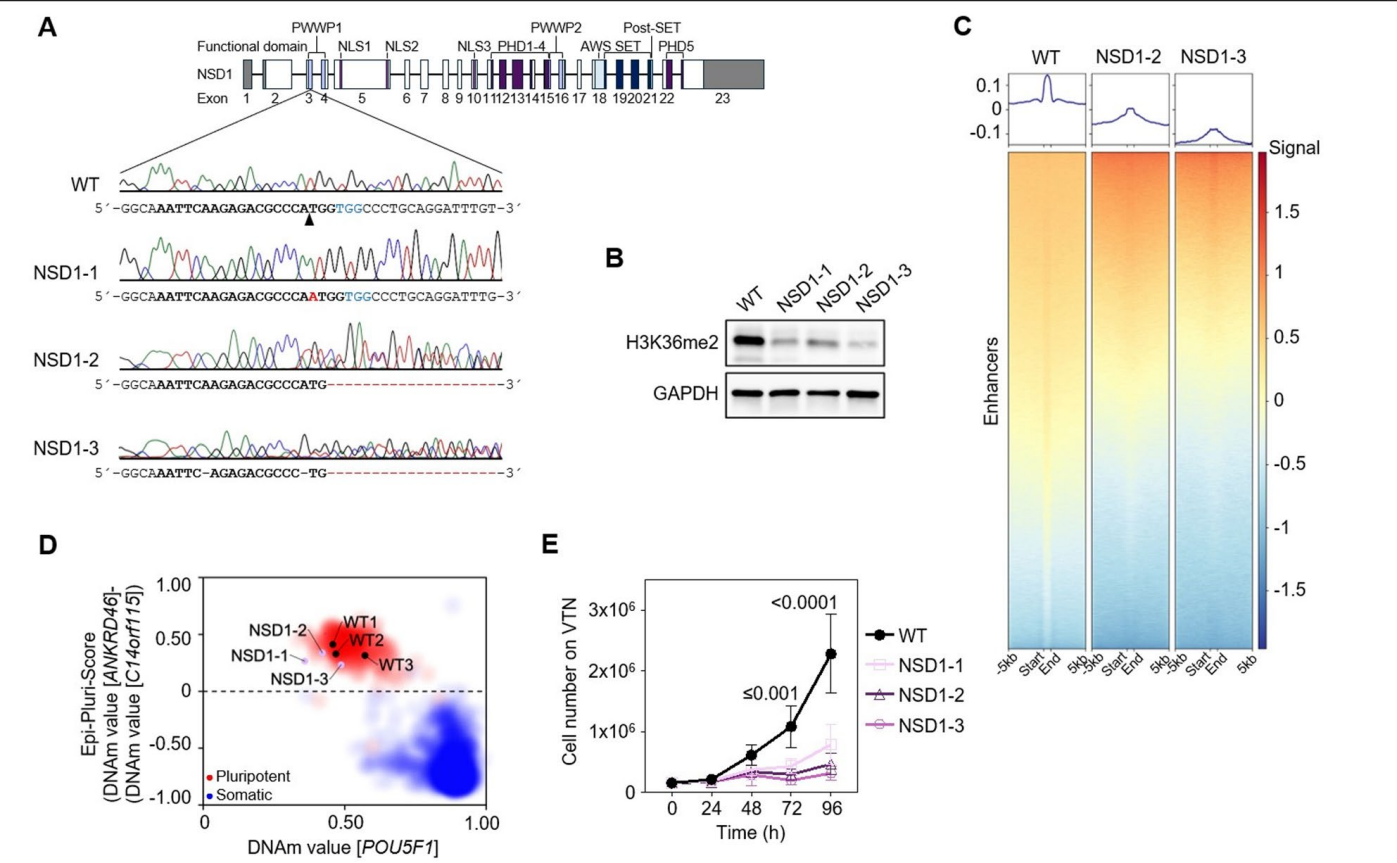
NSD1-KO iPSCs show reduced H3K36me2. (**A**) Domain structure of human *NSD1* and CRISPR/Cas9 strategy targeting exon three of canonical *NSD1*. Three iPS cell lines (NSD1-1, NSD1-2, NSD1-3) with homozygous or heterozygous frameshift mutations were generated. (**B**) Western Blot analysis of WT and the three NSD1-KO iPSC lines using antibodies targeting H3K36me2 and the housekeeping protein GAPDH. (**C**) Heatmap and line plot of H3K36me2 ChIP-seq signal showing enhancer start site end site ± 5 kbp for WT and NSD1-KO clones 2 and 3. (**D**) Epi-Pluri-Score of WT (*n* = 3) and NSD1-KO (*n* = 3) iPSCs. This analysis assesses DNA methylation at three specific CpG sites, including one within the pluripotency gene *POU5F1* (also known as OCT4). Methylation differences in *ANKRD46* and *C14orf115* are combined to calculate the Epi-Pluri-Score. Background dots represent DNA methylation profiles from 264 pluripotent (red) and 1,951 non-pluripotent (blue) cell samples [30]. (**E**) Growth curves of WT and the three NSD1-KO iPSC lines on Vitronectin for 96 h. Each time point was measured in *n* = 3 technical replicates; mean ± standard deviation. Significance was determined by a Two-way ANOVA test, and p-values are depicted

Exon three of the human *NSD1* gene encodes the N-terminal PWWP domain of canonical NSD1 and was targeted in human iPSCs for CRISPR/Cas9 gene editing (Fig. 1A). Three iPSC lines (NSD1-1, NSD1-2, NSD1-3) were generated, and frameshift mutations (NSD1-1) or indels (NSD1-2 and NSD1-3) were confirmed via Sanger Sequencing (Fig. 1A, Supplementary Fig. 1A). While we could not determine the exact sequence of NSD1-2 and NSD1-3 because of large deletions and low quality of sequencing reads, NSD1-1 showed a one base pair insertion that resulted in a frameshift and a premature stop codon (Supplementary Fig. 1A). Off-target editing was not systematically assessed beyond Sanger sequencing of the target locus. To mitigate this concern, all experiments were validated using three independent KO clones with consistent phenotypes, reducing the likelihood that observed effects are driven by clone-specific off-target events. All three generated iPSC lines revealed a loss of NSD1 function, having reduced H3K36me2 in Western Blot and immunophenotypic analysis (Fig. 1B, Supplementary Fig. 1B, C and D). ChIP-Seq analysis for H3K36me2 also showed significantly reduced enrichment in at least two of the three clones (clone 2 and 3) (Fig. 1C, Supplementary Fig. 1E and F), which was especially pronounced in enhancer regions (Fig. 1C). We saw some variation in the residual H3K36me2 signal between the clones, with clone 1 retaining 22%, clone 2, 44.5% and clone 3 retaining just 7.9% of the H3K36me2 (Supplementary Fig. 1C). Despite the significant loss in H3K36me2, NSD1-KO iPSCs retain a pluripotent state which was confirmed by the pluripotency marker OCT4 (Supplementary Fig. 1D) and based on DNA methylation levels by a positive Epi-Pluri-Score, which classifies pluripotent and somatic cells [30] (Fig. 1D). Notably, NSD1-KO iPSCs demonstrated significantly reduced growth compared to WT iPSCs on the common maintenance substrate Vitronectin (VTN) (Fig. 1E). We investigated whether alternative substrates could be used for culture and observed that growth was comparable to WT iPSCs when cultivating NSD1-KO iPSCs on Biolaminin 521 LN (LMN) (Supplementary Fig. 1G), and the cells were grown on LMN for further experiments. Taken together, our NDS1-KO iPSCs show reduced H3K36me2 enrichment in intergenic regions and exhibit significant growth defects.

### NSD1-KO induces global DNA hypomethylation, similar to Sotos syndrome and naïve stem cells

**Fig. 2:**
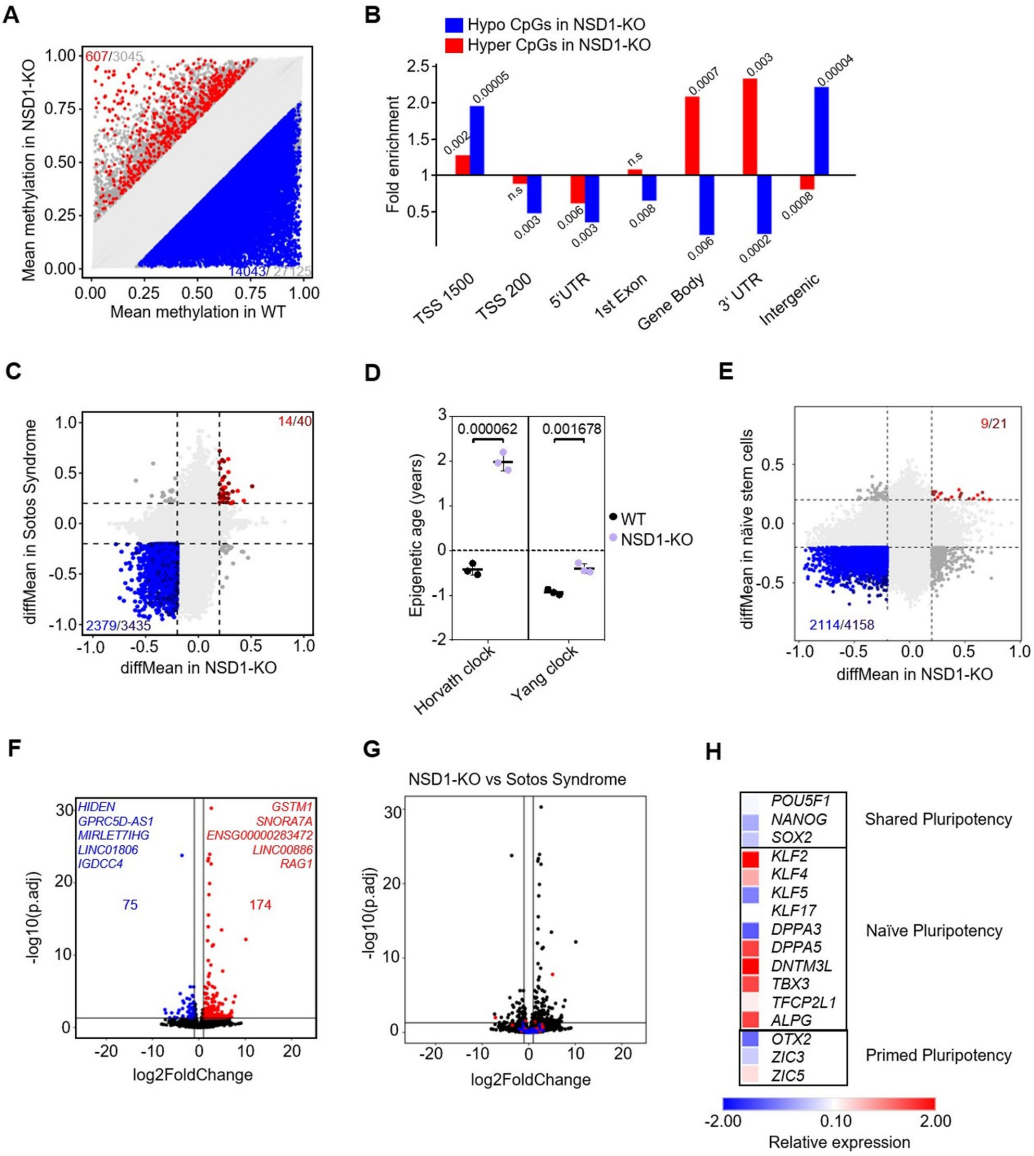
NSD1-KO induces global DNA hypomethylation and modest changes in transcription. (**A**) Scatter plot showing DNA methylation changes between NSD1-KO (*n* = 3) and WT (*n* = 3) with 14,043 hypomethylated sites and 607 hypermethylated sites (difference in mean methylation ≥ 20%, p-value ≤ 0.05). (**B**) Association of hyper- and hypomethylated CpG sites in NSD1-KO iPSCs (*n* = 3) with genomic regions. Hypergeometric tests were used to calculate the significance, and p-values are depicted. (**C**) Comparison of DNA methylation in NSD1-KO iPSCs (*n* = 3) and Sotos syndrome samples (*n* = 3). Mean ∆beta values of significantly different CpGs (difference in mean methylation ≥ 20%, p-value ≤ 0.05) between NSD1-KO and WT are plotted against mean ∆beta values of significantly different CpGs (difference in mean methylation ≥ 20%, p-value ≤ 0.05) between Sotos syndrome and healthy donors. Overlapping hypomethylated (blue) and hypermethylated (red) CpGs are indicated. (**D**) Epigenetic age calculation in WT (*n* = 3) and NSD1-KO (*n* = 3) iPSCs using the Yang clock and the Horvath clock. Age is depicted in years (mean ± standard deviation). Significance was determined by a paired t-test, and p-values are depicted. (**E**) Comparison of DNA methylation in NSD1-KO iPSCs (*n* = 3) and naïve stem cells (*n* = 88) [35]. Mean ∆beta values of significantly different CpGs (difference in mean methylation ≥ 20%, p-value ≤ 0.05) between NSD1-KO and WT are plotted against mean ∆beta values of significantly different CpGs (difference in mean methylation ≥ 20%, p-value ≤ 0.05) between naïve stem cells and primed stem cells. Overlapping hypomethylated (blue) and hypermethylated (red) CpGs are indicated. (**F**) Volcano plot of RNA sequencing analysis of NSD1-KO (*n* = 3) and WT (*n* = 3) iPSCs. 174 genes were significantly upregulated, and 75 genes were significantly downregulated (> 2-fold change, adj. p-value < 0.05). The five genes exhibiting the most pronounced upregulation or downregulation are highlighted. (**G**) Correlation of differentially expressed genes in NSD1-KO iPSCs (*n* = 3) and blood samples (*n* = 3) from patients with Sotos syndrome. The genes upregulated (red dots) and down regulated (blue dots) in Sotos syndrome were overlayed with figure F. No overlap was seen between the two data sets. (**H**) Mean relative expression of genes associated with shared, naïve, and primed pluripotency in NSD1-KO cells (*n* = 3)

To further elucidate the effect of reduced NSD1-mediated H3K36me2 in human iPSCs, we performed Illumina Beadchip-based DNA methylation analysis. WT and NSD1-KO cell lines clustered apart from each other in the MDS plot (Supplementary Fig. 2A). We observed significant DNA hypomethylation at 14,043 CpG sites in NSD1-KO cells (Fig. 2A), consistent for all three clones (Supplementary Fig. 2B). The hypomethylated CpGs mapped largely to intergenic regions (Fig. 2B) and revealed associations with Gene Ontology (GO) terms, such as plasma membrane, monoatomic ion transport, or transporter activity (Supplementary Fig. 2C). In contrast, 607 CpGs were hypermethylated in NSD1-KO cells (Fig. 2A) and mapped to gene body and 3´-UTR regions with less significant GO terms (Fig. 2B, Supplementary Fig. 2D). The overgrowth disorder Sotos syndrome is also characterized by a genome-wide loss of DNA methylation [21]. Brennan et al. identified the hypomethylation at cg07600533 as a diagnostic marker for Sotos syndrome [31], and our results showed significant hypomethylation at cg07600533 (Supplementary Fig. 2E). This prompted us to compare global DNA methylation data in Sotos syndrome and NSD1-KO iPSCs. We used the DNA methylation data from blood of healthy donors and Sotos syndrome patients ([31], GSE191276) and observed that despite arising from different source material (iPSCs *versus* blood), 69.25% (2379 out of 3435) of CpGs significantly hypomethylated in Sotos syndrome were also significantly hypomethylated in NSD1-KO cells (Fig. 2C), indicating that our NSD1-KO cells may partially recapitulate Sotos syndrome DNAm profiles. Epigenetic age was then computed using the Yang clock and the Horvath clock in NSD1-KO cells [32, 33]. We observed a modest but significant epigenetic age acceleration in NSD1-KO iPSCs compared to WT cells (Fig. 2D). To determine whether similar overlaps as with Sotos syndrome samples could be detected in cancers associated with NSD1 mutations, we compared the DNA methylation profiles of head and neck squamous cell carcinoma (HNSC) and lung squamous cell carcinoma (LUSC) samples [34] with NSD1-KO. However, we did not find overlaps between the CpGs hypomethylated in either of the cancer samples and NSD1-KO cells (Supplementary Fig. 2F, G). Global DNA hypomethylation, unrelated to NSD1 function is also observed in naïve stem cells when compared to primed stem cells [27]. We compared DNAm data from naïve stem cells [35] and observed an overlap of 51.1% hypomethylated CpGs between NSD1-KO and naïve stem cells (Fig. 2E).

RNA sequencing analysis of WT and NSD1-KO iPSCs revealed differences in the transcriptome based on the principal component analysis (PCA) (Supplementary Fig. 2H). However, despite the substantial number of differentially methylated CpG sites identified, only a small number of genes showed significantly altered expression in NSD1-KO iPSCs. 174 genes were significantly upregulated, with *GSTM1*, *SNORA7A*, *ENSG00000283472*, *LINC00886*, and *RAG1* representing the five most highly upregulated genes. Among the 75 significantly downregulated genes, long noncoding RNAs (lncRNAs) exhibited the most substantial decrease, with *HIDEN*,* GPRC5D-AS*,* MIRLET7IHG*,* LINC01806* and *IGDCC4*, being the top 5 downregulated genes (Fig. 2F). Since NSD1 has a catalytically independent function as a transcriptional coactivator [15], we combined DNA methylation with transcriptomic data to better understand the regulation of the transcriptome through NSD1-mediated H3K36me2 and subsequent DNA methylation changes. Hypermethylated CpG sites overlapped just with 2 upregulated genes, while the hypomethylated CpGs overlapped with both upregulated (37) and downregulated (29) genes (Supplementary Fig. 2I), indicating a separate function of NSD1 in the regulation of gene expression and DNA methylation. The DNA methylation profile of NSD1-KO cells partially recapitulated that of Sotos syndrome; however, comparative analysis revealed little overlap between genes dysregulated in Sotos syndrome and those in NSD1-KO iPSCs (Fig. 2G). Interestingly, although not significant, genes associated with naïve pluripotency showed higher expression and those associated with primed pluripotency [36] showed lower expression in NSD1-KO cells (Fig. 2H), which, taken together with the DNAm data, may suggest a shift of pluripotency markers in NSD1-KO cells towards a more “naïve-like” state.

### NSD1-KO iPSCs exhibit endodermal and mesodermal differentiation defects

**Fig. 3:**
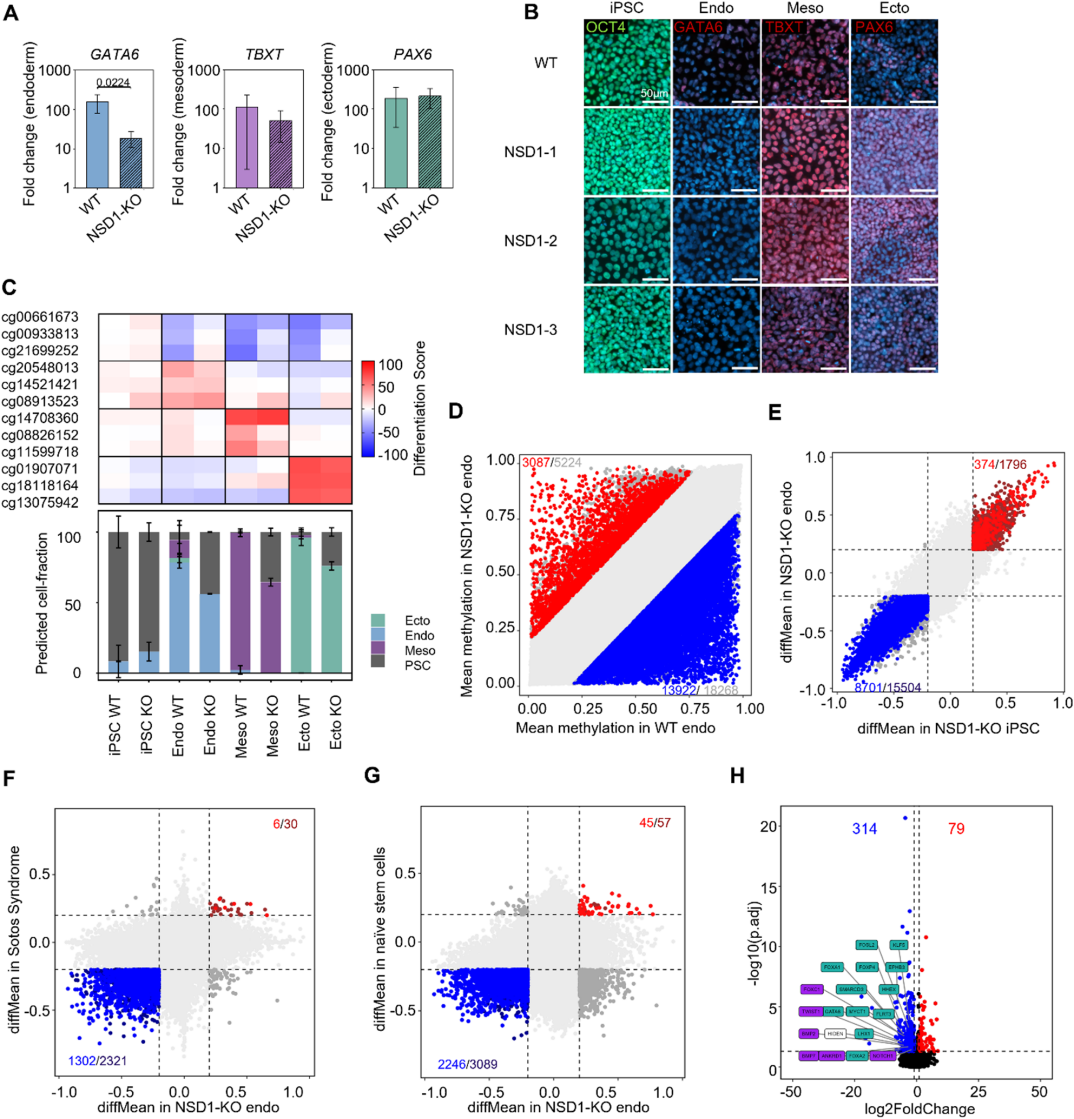
Endodermal differentiation capacity is impaired in NSD1-KO iPSCs. (**A**) qRT-PCR of germ layer-specific markers in endoderm (*GATA6*), mesoderm (*TBXT*), and ectoderm (*PAX6*). The trilineage differentiation was performed in *n* = 3 replicates for WT and the three NSD1-KO clones and normalized to *GAPDH* (mean ± standard deviation). Statistical analysis was performed with an unpaired t-test. P-value is depicted. (**B**) Exemplary immunofluorescence analysis of differentiation into the three germ layers. Antibodies targeting OCT4 (iPSCs), GATA6 (endoderm), TBXT (mesoderm), and PAX6 (ectoderm) were used. *N* = 1 for each clone. Scale bar = 50 μm. (**C**) Differentiation scores of selected CpG sites to evaluate the germ-layer specific differentiation potential of WT (*n* = 3) and NSD1-KO (*n* = 3) iPSCs. Each square represents the mean DNA methylation difference at a germ-layer-specific CpG site relative to reference values of PSCs. Below, the deconvolution shows estimates of the lineage-specific cell fractions during differentiation calculated as shown in [37] (mean ± standard deviation). (**D**) Scatter plot showing DNA methylation changes between NSD1-KO endoderm (*n* = 3) and WT endoderm (*n* = 3) with 13,922 hypomethylated sites and 3,087 hypermethylated sites (difference in mean methylation ≥ 20%, p-value ≤ 0.05). (**E**) Comparison of DNA methylation in NSD1-KO iPSCs (*n* = 3) and NSD1-KO endoderm cells (*n* = 3). Mean ∆beta values of significantly different CpGs (difference in mean methylation ≥ 20%, p-value ≤ 0.05) between NSD1-KO iPSC and WT iPSC are plotted against mean ∆beta values of significantly different CpGs (difference in mean methylation ≥ 20%, p-value ≤ 0.05) between NSD1-KO endoderm and WT endoderm. Overlapping hypomethylated (blue) and hypermethylated (red) CpGs are indicated. (**F**) Comparison of DNA methylation in NSD1-KO endoderm (*n* = 3) and Sotos syndrome (*n* = 3), analogous to E. (**G**) Comparison of DNA methylation in NSD1-KO endoderm (*n* = 3) and naïve stem cells (*n* = 88), analogous to E. (**H**) Volcano plot of RNA sequencing analysis of NSD1-KO endoderm (*n* = 3) and WT endoderm (*n* = 3). 79 genes were significantly upregulated, and 314 genes were significantly downregulated (> 2-fold change, adj. p-value < 0.05). Key endodermal genes (green boxes), mesodermal genes (purple boxes) and the lncRNA HIDEN (clear box) are highlighted (Supplementary Table 3).

Following the detailed characterization of NSD1-KO iPSCs, their differentiation potential towards the three germ layers, endoderm, mesoderm, and ectoderm was investigated. In an undifferentiated state, NSD1-KO iPSCs persisted in an iPSC-like morphology with unaffected expression of the pluripotency marker *POU5F1* (Supplementary Fig. 3A). Expression of germ layer-specific markers, *GATA6* (endoderm), *TBXT* (mesoderm), and *PAX6* (ectoderm) was evaluated in qRT-PCR analysis (Fig. 3A). Upon endodermal differentiation, *GATA6* expression was significantly reduced in NSD1-KO cells compared to the WT, indicating an endodermal differentiation defect. *TBXT* and *PAX6* expression was not significantly different upon mesodermal and ectodermal differentiation, respectively. This trend was corroborated by immunophenotypic analysis, where GATA6 expression was almost absent in NSD1-KO cells while most of the WT cells were GATA6-positive upon endodermal differentiation (Fig. 3B, Supplementary Fig. 3B).

We further validated the differentiation defects with DNA methylation data using the “PluripotencyScreen” [37]. Methylation values of 12 CpG sites – 3 CpGs per germ layer and pluripotent cells – provide a basis for deconvolution analyses to estimate the proportions of cells differentiating into each germ layer. The DNA methylation pattern in NSD1-KO iPSCs resembled an undifferentiated state, similar to WT iPSCs (Fig. 3C). Following directed differentiation, the DNA methylation signature of NSD1-KO cells diverged markedly from WT cells, with a high fraction of differentiated cells still retaining pluripotency signatures, indicating impaired differentiation, particularly towards endodermal and mesodermal lineages (Fig. 3C). When focusing only on pluripotency-associated CpG sites, NSD1-KO cells across all differentiation directions exhibit a DNA methylation pattern closer to a pluripotent state, compared to WT cells, as illustrated by the Pluripotency Score (Supplementary Fig. 3C). Interestingly, differentiation of NSD1-KO iPSCs towards induced mesenchymal stromal cells (iMSCs) was not possible, reinforcing a role of NSD1 in mesodermal differentiation. We further investigated the DNAm and transcription profile of endodermal differentiated NSD1-KO cells. Similar to the iPSCs, we saw a global DNA hypomethylation (13,922 CpGs) compared to WT endodermal cells; (Fig. 3D) 62.4% (8,701 CpGs) of the hypomethylated CpGs were already hypomethylated in NSD1-KO iPSCs (Fig. 3E). We also observed hypermethylation at 3,087 CpGs with 374 CpGs also hypermethylated in NSD1-KO iPSCs (Fig. 3D and E). Comparison of endoderm DNAm profiles with data from Sotos syndrome patients [31] and naïve stem cells [35] also revealed more than 50% overlap between the hypomethylated CpGs (Fig. 3F and G). We further found 79 genes upregulated and 314 genes down regulated in NSD1-KO endodermal cells compared to WT endodermal cells (Fig. 3H). Among the downregulated genes were endodermal genes such as *FOXA1*,* FOXA2*,* GATA6*,* HHEX*,* KLF5* and mesodermal genes such as *NOTCH1*,* BMP2*,* TWIST1* among others (Fig. 3H, Supplementary Table 3). Taken together, our results indicate that NSD1 is an essential factor for early germ layer differentiation, especially in the endodermal and mesodermal lineages.

### The role of NSD1 in endodermal differentiation

**Fig. 4:**
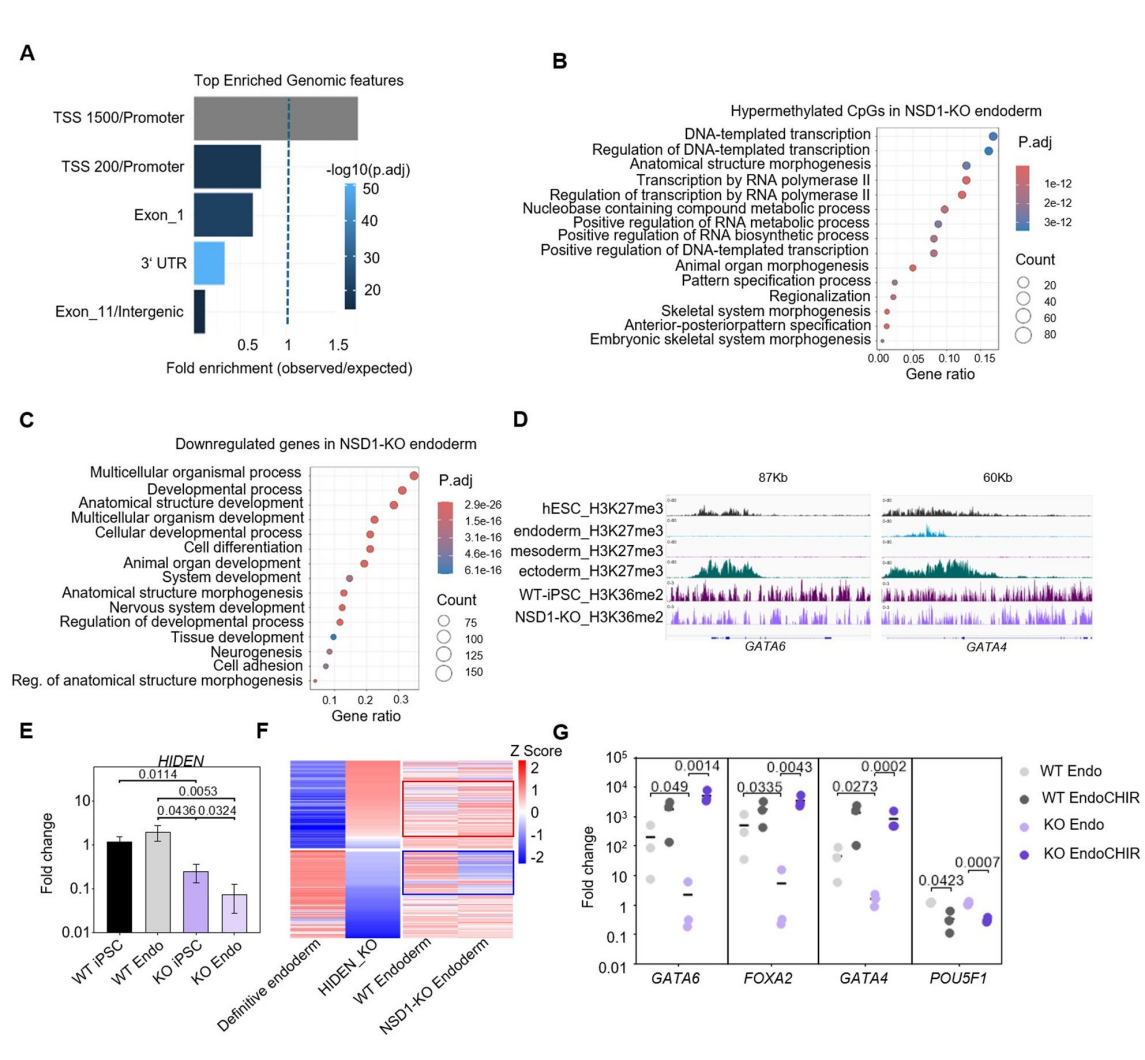
NSD1-mediated early differentiation is induced via lncRNA HIDEN. (**A**) Association of hypermethylated CpG sites in NSD1-KO endoderm (n=3) with genomic regions. (**B**) Gene set enrichment analysis of hypermethylated CpG sites in NSD1-KO endoderm (n=3). (**C**) Gene set enrichment analysis of downregulated genes in NSD1-KO endoderm (n=3). (**D**) Visual representation of H3K27me3 enrichment (ENCODE data sets) at the GATA6 and GATA4 gene locus in hESCs, endoderm, mesoderm, and ectoderm, and H3K36me2 enrichment in WT and NSD1-KO iPSCs. (**E**) Gene expression (mean ± standard deviation) of HIDEN in undifferentiated WT and NSD1-KO iPSCs and after endodermal differentiation (all n=3 biological replicates). Fold changes were normalized to GAPDH. Statistical analysis was performed with an unpaired t-test. P-values are depicted. (**F**) Comparative analysis of the top 100 differentially expressed genes in endodermal differentiated HIDEN-KO hPSCs [29] (n=3) and endodermal differentiated WT (n=3) and NSD1-KO (n=3) iPSCs. The red and blue boxes indicate the subset of up and downregulated genes in HIDEN-KO endoderm that are also analogously regulated in NSD1-KO endoderm. (**G**) Expression analysis of endodermal markers GATA6, FOXA2, and GATA4 and pluripotency marker POU5F1 in endodermal differentiated WT (n=3) and NSD1-KO (n=3) iPSCs without and with treatment with the WNT agonist CHIR. Mean fold change compared to undifferentiated iPSCs, after normalization with GAPDH is plotted. Statistical analysis was performed with an unpaired t-test. p-values are depicted.

It has been previously reported that the loss of NSD1 leads to mesodermal defects [14]. We therefore decided to further investigate the role of NSD1 in endodermal differentiation. NSD1 regulates the binding of DNMT3A to intergenic regions and loss of NSD1 had been shown to lead to redistribution of DNMT3A and hence DNAm to gene promoters and gene body through the existing H3K36me3 mark [12, 15, 21, 38]. Indeed, we found an overrepresentation of hypermethylated CpGs at gene promoter regions in endodermal differentiated NSD1-KO cells (Fig. 4A), which was not observed for hypomethylated CpGs (Supplementary Fig. 3D). Gene Set Enrichment analysis (GSEA) showed that the hypermethylated CpGs were associated with genes involved in transcription, pattern specification, and organ morphogenesis (Fig. 4B). Similar terms, along with cell differentiation, development and cell adhesion were also enriched in genes downregulated in endodermal NSD1-KO cells (Fig. 4C), which were not observed for hypomethylated CpGs or upregulated genes (Supplementary Fig. 3E and F). This indicates that the endodermal defect may in part be caused by the downregulation of differentiation genes as a result of hypermethylation of the promoters. Analysis of published ENCODE H3K27me3 profiles from hESCs, endoderm, mesoderm, and ectoderm [ENCSR085CKM (H1 ESCs), ENCSR832CNQ (Endoderm), ENCSR000DSL (Mesoderm) and ENCSR690GLT (Ectoderm)], implied that the key endodermal regulators including GATA6, GATA4 are polycomb targets (Fig. 4D). This indicates that the loss of NSD1-mediated H3K36me2 from these regions could facilitate polycomb mediated H3K27me3 deposition at endodermal genes, repressing them, leading to the endodermal defect.

While NSD1-KO leads to modest changes in transcription, one of the most downregulated genes in NSD1-KO iPSCs and after endodermal differentiation was the long non-coding RNA (lncRNA) *HIDEN* (ENSG00000253507; Transcript ID ENSG00000253507.5) (Figs. 2F and 3H). Lu et al. reported that *HIDEN* is important for mRNA stability of the WNT receptor FZD5, positioning it as a crucial modulator for endodermal differentiation. Notably, knockout of *HIDEN* resulted in impaired endodermal differentiation capacity of human pluripotent cells [29].

We confirmed the reduction of *HIDEN* expression in NSD1-KO by qRT-PCR and observed significantly reduced expression compared to WT cells in the pluripotent state and after endodermal differentiation (Fig. 4E). Further investigating the mechanisms of NSD1-mediated regulation of *HIDEN* expression, we found that the *HIDEN* region exhibited an enrichment in H3K36me2 in WT iPSCs, which was significantly diminished in NSD1-KO cells already in the pluripotent state, especially in clones 2 and 3 (Supplementary Fig. 3G). CpG sites surrounding *HIDEN* promoter region and gene body (cg25940523, cg21410491, cg01065516, cg26980111, cg02642958), however, did not show a significant change in DNA methylation levels (Supplementary Fig. 3H). We investigated whether HIDEN-KO definitive endoderm cells [29] and NSD1-KO endodermal cells would exhibit similar gene expression profiles by comparing RNA sequencing data (GSE188501) and observed that a subset of the top 100 genes up- or downregulated in HIDEN-KO endoderm were also similarly up-or down regulated in NSD1-KO (Fig. 4F), reinforcing the possible role of HIDEN in NSD1-mediated endodermal differentiation.

Previous studies indicated that treatment with the WNT agonist CHIR was able to rescue the endodermal defect caused by HIDEN-KO [29]. Interestingly, we were also able to recover endodermal differentiation as measured by significant upregulation of *GATA6*, *FOXA2*, and *GATA4* upon treatment of NSD-1 KO cells with CHIR (Fig. 4G). This is consistent with our hypothesis that NSD1-mediated H3K36 dimethylation regulates *HIDEN* expression and thereby regulates endodermal germ-layer differentiation of human iPSCs.

## Discussion

Human iPSCs provide unparalleled potential for the modeling of diseases, especially developmental disorders [39]. We attempted to generate human iPSCs functionally deficient in NSD1, the major histone methyltransferase responsible for establishing the intergenic H3K36me2 modification. While frameshift (NSD1-1) and indel mutations (NSD1-2 and 3) were confirmed by Sanger sequencing, we did not see a complete loss of the H3K36me2 mark. We could not demonstrate the loss of NSD1 at protein level due to nonspecific binding of the NSD1 antibodies we tested. However, we saw significant reduction of H3K36me2 levels with western blot, immunofluorescence, and ChIP-Seq. While NSD1 is the predominant H3K36me2 transferase, studies have identified other NSD family proteins, such as NSD2 and NSD3, that establish this modification [40]. The activity of NSD2 and 3 may explain the residual H3K36me2 we observe in our cells and suggest that our experiments likely underestimate the full phenotypic impact of complete NSD1 loss. H3K36me2 ‘reader’ proteins such as LEDGF and MSH6 may still be recruited and function at the residual methylation sites, masking the true consequences of total ablation [41]. As all three clones behaved consistently across the assays we performed, the phenotypic effects were most likely a result of the NSD1 loss and not off-target effects. ChIP-Seq analysis revealed a significant reduction of the H3K36me2 mark in NSD1-KO cells, especially at enhancers. This is in line with previous studies that implicate NSD1 in enhancer function [16, 25]. While NSD1-deficient mice and mouse ESC lines have been generated before [14–16, 25, 31], we report novel human iPSC lines with functionally deficient NSD1, which may be used as model systems to further study the acquisition of the developmental defects occurring in overgrowth disorders caused by NSD1 mutations.

As also seen in Sotos syndrome, NSD1-KO leads to severe DNA hypomethylation but only a modest change in transcription [18], with more than half the CpGs hypomethylated in Sotos syndrome also hypomethylated in NSD1-KO iPSCs. There was little overlap between the differentially expressed genes, however, indicating that the DNA methylation profiles resulting from a loss of NSD1 may already be partially established early in development, and the gene expression patterns may be differentiation specific. The apparent discordance may also result from the differences in tissues tested, developmental timing, or compensatory transcription mechanisms from the residual H3K36me2. Furthermore, we observed little overlap with DNAm changes in cancer with inactivating NSD1 mutations, indicating that our cell lines may partially recapitulate developmental phenotypes rather than those related to cancer progression. Previous reports have suggested that growth disorders such as Tatton-Brown-Rahman syndrome and Sotos syndrome are associated with accelerated epigenetic age [22]. Our study revealed a small but significant increase in epigenetic age in NSD1-KO iPSCs. While it should be noted that iPSCs are epigenetically rejuvenated and may not accurately reflect an acceleration of epigenetic age, the perceived acceleration in overgrowth disorders may also be a readout of global hypomethylation, and functional studies may determine whether the overgrowth phenotype corresponds to functional aging.

One of the key findings of our study is that NSD1-KO iPSCs retain pluripotency markers. In fact, we see DNA methylation levels similar to pluripotent cells even after differentiation in the three germ layers, resulting in a higher pluripotency score. A previous report has suggested that H3K36me is a barrier to reprogramming. Suppression of H3K36 methylation enhanced the reprogramming of mouse embryonic fibroblasts (MEFs), associated with a PRC2-dependent silencing of mesenchymal genes and increased expression of epithelial and pluripotency genes [42, 43]. We observe a similar push towards pluripotency upon loss of NSD1. Furthermore, we see DNAm and expression profiles partially reminiscent of naïve stem cells, which may explain the retention of pluripotency markers even after differentiation. In both mouse and human systems, naïve pluripotent cells are biased away from definitive endoderm and mesoderm and toward extraembryonic fates [44, 45]. Our data suggest that NSD1 loss may alter the pluripotent ground state sufficiently to impair germ layer specification without achieving full naïve conversion.

Additionally, we observe defects in the differentiation potential in NSD1-KO cells, especially along the endoderm and mesoderm lineages. Differentiation of our NSD1-KO iPSCs into iMSCs was impaired, consistent with previously reported mesodermal defects in developing NSD1^−/−^ mice [14]. However, we cannot determine whether NSD1-KO iPSCs fail to form mesoderm entirely (early defect) or form mesoderm but cannot commit to the mesenchymal lineage (late defect). NSD1-KO in mouse ESCs exhibited differentiation defects in all three germ layers, and auxin-degron-based NSD1 degradation showed defects in cardiomyocyte differentiation and neural specification in mESCs [15, 16]. Furthermore, NSD1 deficiency in mesenchymal progenitors lead to impaired chondrogenic differentiation and skeletal growth [46]. Endoderm specific genes were not only downregulated in NSD1-KO cells, also DNA hypermethylation was seen at the promoters of genes involved in differentiation. Loss of H3K36me2 has been reported to increase H3K27me3 at target sites [23, 47]. As polycomb proteins play a major role in regulating endodermal differentiation [48, 49], with key endodermal genes being polycomb targets (Fig. 4D), our data suggest that the endodermal defect may result from polycomb-mediated repression of endodermal genes in addition to DNA hypermethylation at promoters.

We further identify the endoderm-specific desert lncRNA *HIDEN* as being one of the most highly repressed gene upon NSD1-KO in undifferentiated as well as endodermal differentiated state and demonstrate that NSD1 may regulate endodermal differentiation mediated by *HIDEN* and the WNT signaling pathway. While H3K36me2 levels decrease upstream and in the intronic regions of *HIDEN*, the CpGs do not show a change in methylation. This indicates the NSD1 regulated *HIDEN* expression via mechanisms independent of DNA methylation and may rely on crosstalk between H3K36me2 and other histone modifications for regulating expression. Testing this model will require direct perturbation experiments such as targeted demethylation or methylation of the HIDEN locus combined with chromatin profiling for other histone modifications (e.g., H3K27me3, H3K4me1/3, H3K36me3) to identify the actual downstream effector mechanism. Genes misregulated in HIDEN-KO cells show partial overlap with those in NSD1-KO cells. While this overlap is consistent with HIDEN acting downstream of NSD1 for a subset of targets, the substantial non-overlapping gene sets indicate that NSD1 and HIDEN could also regulate distinct transcriptional programs. Definitive determination of HIDEN’s role will require rescue experiments, RNA-IP, and mechanistic studies of *FZD5* transcript stability.

Integrating our results with previous reports that implicate NSD1 in regulating cell fate determination through enhancer binding and bivalent gene expression [16, 25, 31] reveals a dual mechanism of NSD1 function. On one hand, NSD1 governs the transcription of developmental lncRNA such as *HIDEN* and potentially other endodermal genes by preventing these genomic regions from undergoing polycomb-mediated silencing. This helps maintain appropriate gene expression profiles that are necessary for proper cell fate determination. On the other hand, NSD1 may also be required for preserving cell-type-specific DNA methylation profiles. In the absence of NSD1, global DNA hypomethylation occurs, particularly at intergenic regions in iPSCs. This global loss is accompanied by a redistribution of methylation at the promoters of differentiation genes. Together, these epigenetic alterations may ultimately lead to the lineage-specific defects observed both in our cellular models and in individuals with Sotos syndrome. NSD1-knockout human iPSC lines may therefore provide a valuable system for investigating early differentiation defects, thereby offering insights into how lineage-specific phenotypes arise in overgrowth disorders.

## Methods

### Cell culture and directed differentiation

Three independent human induced pluripotent stem cell lines were reprogrammed from bone marrow-derived mesenchymal stromal cells by episomal plasmids – hPSCreg: UKAi009-A (wildtype (WT)1), UKAi010-A (WT2), and UKAi011-A (WT3). All samples were taken after informed and written consent using guidelines approved by the Ethics Committee for the Use of Human Subjects at the University of Aachen (permit number: EK128/09). All methods were performed in accordance with the relevant guidelines and regulations. Maintenance culture of WT iPSCs was performed on tissue culture plastic (TCP) coated with vitronectin (VTN, 0.5 µg/cm^2^; Stem Cell Technologies, Vancouver, Canada) or with Biolaminin 521 LN (LMN, 0.9 µg/cm^2^, BioLamina, Sundbyberg, Sweden) for NSD1-KO iPSCs. IPSCs were cultured in StemMACS iPS-Brew XF (Miltenyi Biotec GmbH, Bergisch Gladbach, Germany). For directed differentiation towards the three germ layers, endoderm, mesoderm, and ectoderm, the STEMDiff™ Trilineage Differentiation Kit (Stem Cell Technologies, Vancouver, Canada) was used according to the manufacturer’s instructions. Differentiation into endodermal and mesodermal direction lasted for 5 days and into ectodermal direction for 7 days. As an undifferentiated control, iPSCs were maintained in StemMACS iPS-Brew XF for 5 days. Endodermal rescue experiments were performed by treatment of cells with endoderm differentiation medium containing 2 µM CHIR99021 (CHIR, Tocris). Differentiation towards induced mesenchymal stromal cells (iMSCs) was performed according to a protocol from [26]. The differentiation was initiated by switching from iPS-Brew XF to DMEM low glucose supplemented with 10% human platelet lysate when a confluency of 60–70% was reached. Cells were maintained for 7 days (P0) and then passaged to 0.1% gelatin-coated TCP (P1). Since the NSD1-KO cells were not viable following passaging, the differentiation was not continued.

### CRISPR/Cas9 mediated NSD1 modification

For the generation of NSD1-KO cell lines, WT1 iPSCs were transfected with a CRISPR/Cas9 nuclease approach. The guide RNA was designed to target exon 3 within the human NSD1 gene (ENSG00000165671, gRNA sequence: AATTCAAGAGACGCCCATGG). The ribonucleoprotein (RNP) was assembled using Alt-R CRISPR/Cas9 crRNA, Alt-R CRISPR/Cas9 tracrRNA, and Alt-R HiFi Sp. Cas9 Nuclease (all IDT, Coralville, USA), and transfected into WT1 iPSCs using the NEON transfection system (1300 V, 30 ms pulse width, 1 pulse; Thermo Fischer Scientific, Waltham, USA). Following the electroporation, transfected cells were seeded on a coating of Biolaminin 521 LN in StemMACS iPS-Brew XF, supplemented with Rho-associated protein kinase (ROCK) inhibitor Y-27,632 (10 µM, Abcam, Cambridge, UK) and 1x CloneR (Stem Cell Technologies, Vancouver, Canada). Four days later, cells were singularized and seeded on mouse embryonic fibroblasts (MEFs). After eight days, individual iPSC colonies were picked and seeded on MEFs for an additional passage and subsequently expanded in a feeder-free environment on vitronectin-coated plates. Expanded clones were screened for mutations in exon 3 via Sanger Sequencing (Eurofins Genomics, Ebersberg, Germany; sequencing primer: AAAGTCTACGCCACTGAAGTATG). The culture of the three NSD1-KO clones was changed to LMN coating, after a significant increase in the viability of NSD1-KO iPSCs on LMN was seen.

### Growth curves

Equal numbers of cells in single-cell suspensions (150,000 cells; WT1, NSD1-KO clone 1, 2, 3) were seeded onto TCP coated with either VTN or LMN. For cell counting, cultures were dissociated using Accutase (Stem Cell Technologies, Vancouver, Canada), collected by centrifugation, and counted using the Countess 3 automated cell counter (Thermo Fischer Scientific, Waltham, USA). Cell counts were determined every 24 h over 4 days in triplicates. Significance was determined by a Two-way ANOVA test.

### Western blot

Total cell extracts from iPSCs were prepared in cold lysis buffer (RIPA buffer, 50 mM NaF, 1 mM Na_3_VO_4_, and 7× complete mini protease inhibitor). The protein concentration was determined by Bradford protein assay, measured in a photometer (Infinite 200 PRO, Tecan Trading AG, Switzerland). Of each sample, 15 µg protein was incubated in 4x SDS Protein Sample Buffer (50 mM Tris/HCl pH 6.8, 2% SDS, 0.01% Bromophenol blue, 2.5% β-mercaptoethanol and 10% glycerol) for 5 min at 99 °C. The samples were separated in 12% Mini-PROTEAN TGX Precast Protein Gels (Bio-Rad, München, Germany) and transferred to a polyvinylidene fluoride membrane (PVDF; Merck Millipore, Burlington, USA). The membrane was blocked in 4%w/v bovine serum albumin (BSA) for one hour and then incubated in primary antibodies against H3K36me2 and GAPDH overnight at 4 °C (all antibodies indicated in Supplementary Table 1). Incubation with secondary antibodies was done for 1 h at room temperature (RT), and protein bands were visualized using a ChemiDoc XRS+ (Bio-Rad Laboratories, Hercules, USA). The quantification of the Western Blot was conducted with the help of FIJI/ImageJ [50].

### Immunophenotypic analysis

Fixation of the cells was performed with 4% paraformaldehyde for 20 min. Fixed cells were treated with PBS containing 1%w/v BSA and 0.1%v/v Triton-X-100 (Bio-Rad, München, Germany) for 1 h at 4 °C, and then incubated in primary antibodies (Supplementary Table 1) overnight at 4 °C. Secondary antibody staining was performed at RT for 1 h, and thereafter, nuclei were counterstained with Hoechst 33,342. Fluorescence microscopy was performed with a Zeiss Axio Observer (Carl Zeiss, Oberkochen, Germany) using 20x or 40x or 63x objectives.

### Semi-quantitative reverse-transcriptase PCR

Total RNA was extracted with the NucleoSpin RNA Plus Kit (Macherey-Nagel, Düren, Germany) and quantified with a NanoDrop 2000 spectrophotometer (Thermo Scientific, Waltham, USA). Isolated RNA was converted into complementary DNA (cDNA) using the High-Capacity cDNA Reverse Transcription Kit (Applied Biosystems, Waltham, USA). Cycling conditions included the following steps: Primer annealing: 10 min at 25 °C; Reverse transcriptase: 120 min at 37 °C; Enzyme deactivation: 5 min at 85 °C before storing samples at 4 °C. To analyze the gene expression of target genes, a semi-quantitative real-time PCR (qRT-PCR) was performed, using TaqMan gene expression master mix and gene-specific TaqMan assays (Supplementary Table 2). The qRT-PCR was performed in a StepOnePlus Real-Time PCR cycler (UNG incubation: 2 min at 50 °C; Enzyme activation: 10 min at 95 °C; Denaturation: 15 s at 95 °C & Annealing & elongation: 60 s at 60 °C for 40 cycles). For analysis of aberrant endodermal differentiation, qRT-PCR was performed using iTaq Universal SYBR Green Supermix (Bio-Rad, München, Germany) and primers mentioned in Supplementary Table 2 in a CFX Duet Real-Time PCR System (Bio-Rad, München, Germany; Enzyme activation: 30 s at 95 °C; Denaturation: 3 s at 95 °C & Annealing & elongation: 20 s at 60 °C for 40 cycles). All reactions were pipetted in technical duplicates. After performing the qRT-PCR, results were analyzed with the ∆∆Ct method, normalizing to the housekeeping gene *GAPDH*. Statistical analysis was performed with an unpaired t-test.

### Transcriptomic analysis

Total RNA from iPSCs was isolated with the NucleoSpin RNA Plus Kit. For each sample, 700 ng RNA were sent to Life&Brain GmbH (Bonn, Germany), where library preparation (QuantSeq 3′-mRNA) and RNA-sequencing were performed on a NovaSeq6000 sequencer. Sample preprocessing and quality control were performed using nextflow pipeline for RNAseq version 3.12.0: quality of FASTQ files was quantified using FastQC, and adaptor sequences and low-quality reads were trimmed using Trim Galore!. Alignment of the reads was done using STAR (hg38 genome build), and transcript quantification was performed using Salmon. The resulting count matrices were normalized by size factor and dispersion using the DESeq2 package in R. Differential gene expression analysis between NSD1-KO and WT samples was performed using Negative Binomial GLM fitting and Wald-test, and p-values were adjusted with the Benjamini-Hochberg procedure. Genes with adjusted p-value < 0.05 and absolute log2fold change > 1 were considered as differentially expressed. Graphical representation of gene expression differences was done using the Z-Score, computed from normalized counts according to the formula: (counts – mean(counts))/sd(counts). A list of signature genes for Sotos Syndrome was obtained from [18] (GSE191276). A list of genes misregulated in HIDEN-KO was obtained from [29] (GSE188501).

### Pyrosequencing

Genomic DNA (gDNA) was isolated from differentiated and undifferentiated wildtype and NSD1-KO iPSCs using the NucleoSpin Tissue Kit (Macherey-Nagel, Düren, Germany) and quantified with a NanoDrop 2000 spectrophotometer (Thermo Scientific, Waltham, USA). 500 ng gDNA were bisulfite converted with the EZ DNA methylation Kit (Zymo Research), and target sequences were PCR amplified using the Pyromark PCR Kit (Qiagen) with 2.5 mM Mg^2+^ and a primer concentration of 0.3 µM (Supplementary Table 2; Activation: 15 min at 95 °C; Denaturation: 30 s at 94 °C & Annealing: 30 s at 56 °C & Extension: 30 s at 72 °C for 40 cycles; Final extension: 10 min at 72 °C; Storage at 4 °C). Pyrosequencing was performed on a Q48 pyrosequencer (Qiagen) and analyzed using the Pyromark Q48 autoprep software (Qiagen). Pluripotency score, differentiation scores, and deconvolution were analyzed from the DNA methylation values as described before [37]. Depicted differentiation scores and deconvolution show the mean of three biological replicates.

### DNA methylation profiling

Genomic DNA was isolated from three wildtype and NSD1-KO iPSC lines using the NucleoSpin Tissue Kit (Macherey-Nagel, Düren, Germany), bisulfite converted and analyzed with the Illumina human EPIC methylation microarray version 2 (EPIC v2; all analyzed at Life and Brain GmbH, Bonn, Germany). For comparison of NSD1-KO with Sotos syndrome, LUSC and HNSC, public datasets from [31, 34] were used. Initial quality control of the DNA methylation data was conducted using the minfi package (v1.50.0) [51]. The preprocessing, including dye bias correction, quality mask filtering, NOOB normalization, and detection p-value calculation, was performed using the SeSAMe package (v1.22.2) [52]. We excluded CpG probes that failed in 10% or more of the samples, non-CpG probes, and probes on the X and Y chromosomes. The data was converted into a GenomicRatioSet for further analysis with minfi-based functions. BetasCollapseToPfx function from Sesame was used to collapse betas by averaging probes with common probe ID prefix. For the multidimensional scaling (MDS) plots and differential methylation analysis, we used Limma (v3.60.0) [53], considering probes with a mean beta value difference of ≥ 0.2 and adjusted p-values (Benjamini-Hochberg) of ≤ 0.05 as significant. Heatmap was generated using the ComplexHeatmap package (v2.20.0), employing Pearson correlation as the distance measure and the “ward.D2” clustering method. Gene ontology analysis was conducted with the missMethyl R package (v1.38.0) [54]. The R packages: watermelon, ggplot2, ggrepel, ggbeeswarm, reshape2, ggExtra, gprofiler2, and ComplexHeatmap were used for graphical presentation. Epigenetic age was calculated using the Horvath multi-tissue clock [32] or the ‘Yang clock’ reported in [33]. Previously published data (GSE57318, GSE65214, GSE208299, GSE288982, GSE179473, GSE128125, GSE128186, GSE224697) and unpublished data from our lab were integrated to generate DNAm profiles of naïve pluripotent stem cells.

### Chromatin immunoprecipitation sequencing

iPSCs were fixed with 1% formaldehyde and sonicated using the Covaris M200 (75 W, 10%, 200 cycles/burst) for 30 min to obtain chromatin fractions ranging from 200 bp-1kbp. Sonication was performed by the Genomics Facility, IZKF, University Hospital, Aachen. 20 µL of fragmented chromatin were incubated overnight with 4 µL H3K36me2 antibody – ChIP Grade (abcam #ab9049) and 9 µL Blocker (Active Motif #53042) in ChIP Buffer supplemented with PIC (Active Motif #53042). Reactions were incubated with protein-G Agarose Beads (Active Motif #53042). The ChIP protocol was conducted according to the manufacturer’s recommendations (Active motif #53042). Library preparation and ChIP sequencing were conducted by Cologne Centre for Genomics. Fastq files were analyzed using the nextflow pipeline (nf-core/chipseq version 2.1.0). BigWig files, for each ChIP sample normalized to its input, were created from sortedBam files generated by nextflow (SAMtools), using bamCompare from deepTools (binSize 50). BigWig tracks were visualized using IGV [55, 56]. Using computeMatrix from deepTools, signal matrices were generated and then used to create line plots and heatmaps. GeneHancer BED GRCh38 file was obtained from GeneCards.org [57]. For the analysis of H3K27me3, we downloaded the call sets from the ENCODE portal with the following identifiers: ENCSR085CKM (H1 ESCs), ENCSR832CNQ (Endoderm), ENCSR690GLT (Ectoderm) and ENCSR000DSL (Mesoderm).

## Supplementary Information

Below is the link to the electronic supplementary material.

Supplementary Material 1

## Data Availability

All the data generated in the study is published in GEO and can be accessed using the following accession numbers: GSE298829 (EPIC array), GSE298830 (RNA-Seq), and GSE298831 (ChIP-Seq).
