# Supplementary material for: NSD1 governs H3K36me2-mediated DNA methylation and drives endo-mesodermal differentiation of human iPSCs
Source: Clin Epigenetics. 2026 May 20;18:82. doi: 10.1186/s13148-026-02162-5 (PMC13188773; doi:10.1186/s13148-026-02162-5)
Supplement: Supplementary file 1 — Supplementary Material 1 [file 13148_2026_2162_MOESM1_ESM.docx]

Supplemental Material


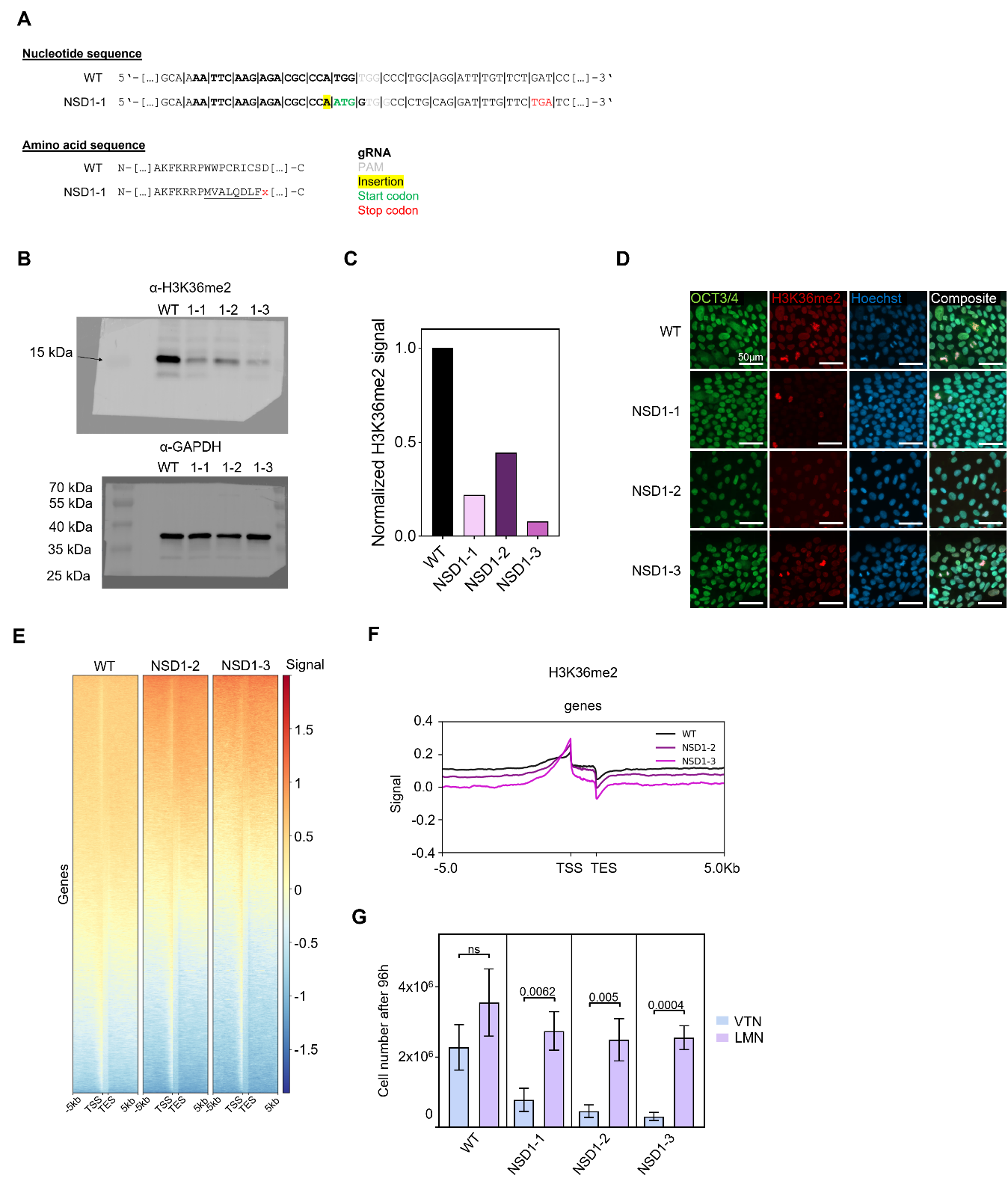


## Supplementary Figure 1: Generation of NSD1-KO iPSCs

**(A)** Sanger sequencing results for NSD1-1 showing the 1 bp insertion and the premature stop codon in the nucleotide sequence, which leads to a termination of the amino acid sequence. **(B)** Uncropped Western Blot images of WT and the three NSD1-KO iPSC lines using antibodies targeting H3K36me2 and the housekeeping protein GAPDH. **(C)** Quantification of the H3K36me2 signal compared to the GAPDH signal from the Western Blot shown in Figure 1B. **(D)** Exemplary immunophenotypic analysis of WT and NSD1-KO iPSCs. Cells were stained with an OCT3/4 and H3K36me2 antibody and nuclei were counterstained with Hoechst. Staining was performed in quadruplicates. Scale bar = 50 µm. **(E)** Heatmap showing H3K36me2 ChIP-seq signal over transcription start site (TSS) and transcription end site (TES) ±5 kbp for WT and NSD1-KO clones 1-2 and 1-3. **(F)** Line plot showing H3K36me2 ChIP-seq signal over transcription start site (TSS) and transcription end site (TES) ±5 kbp for WT and NSD1-KO clones 1-2 and 1-3. **(G)** Quantification of the total cell number (mean ± standard deviation) of WT and NSD1-KO iPSCs growing on vitronectin or laminin after 96h. Cell count was measured for n=3 technical replicates. Statistical analysis was performed using an unpaired t-test, and p-values are depicted.

##
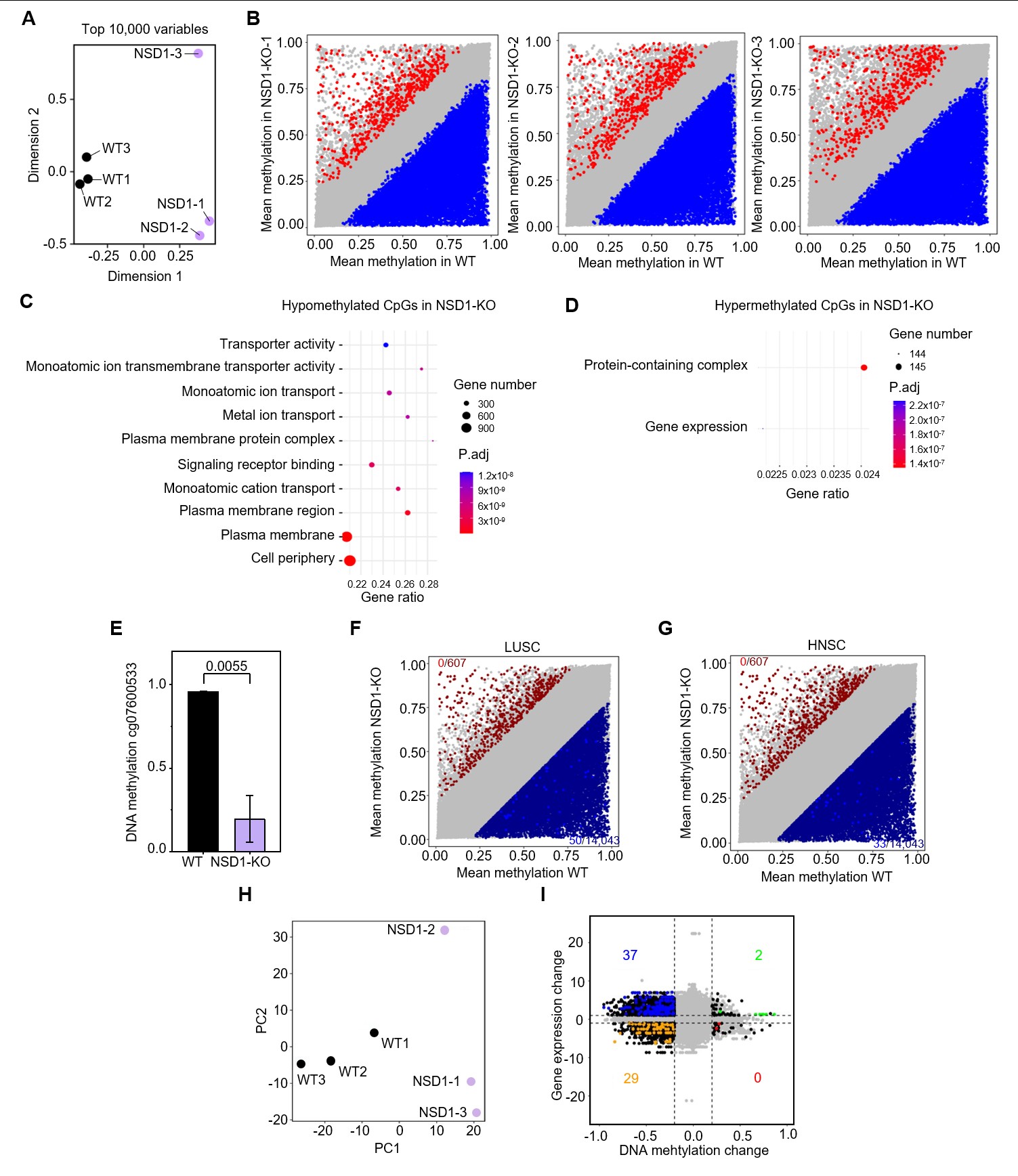
Supplementary Figure 2: DNA methylation and gene expression analysis of NSD1-KO cells

**(A)** MDS plot of the top 10,000 variables of DNA methylation data from three WT and three NSD1-KO iPSC clones. **(B)** Scatter plot showing DNA methylation changes between individual clones of NSD1-KO and WT (n=3; difference in mean methylation ≥20%, p-value≤0.05). **(C)** Gene set enrichment analysis of hypomethylated CpG sites in NSD1-KO (n=3) indicates significant enrichment of genes associated with the plasma membrane and ion transport. **(D)** Gene set enrichment analysis of hypermethylated CpG sites in NSD1-KO (n=3). **(E)** Bar plot showing the DNA methylation of the Sotos syndrome diagnostic CpG site in WT (n=3) and NSD1-KO (n=3) iPSCs (mean ± standard deviation). Statistical analysis was performed using an unpaired t-test, and p-value is depicted. **(F-G)** Comparison between differentially methylated CpGs in NSD1-KO iPSCs (n=3) and LUSC (n=10) (F) and HNSC (n=44) (G). Only 50 and 33 CpGs hypomethylated in NSD1-KO were also hypomethylated in LUSC and HNSC respectively (light blue) (difference in mean methylation ≥20%, p-value≤0.05). No overlaps were detected between the hypermethylated CpGs. **(H)** PCA of RNA sequencing results of three WT and three NSD1-KO iPSC clones. **(I)** Association between DNA methylation levels (difference in mean methylation ≥20%, p-value≤0.05) and corresponding gene expression (>2-fold change, adj. p-value<0.05) in WT (n=3) and NSD1-KO (n=3) iPSCs. Each data point represents a gene-CpG pair, with potential duplication of genes and CpGs.


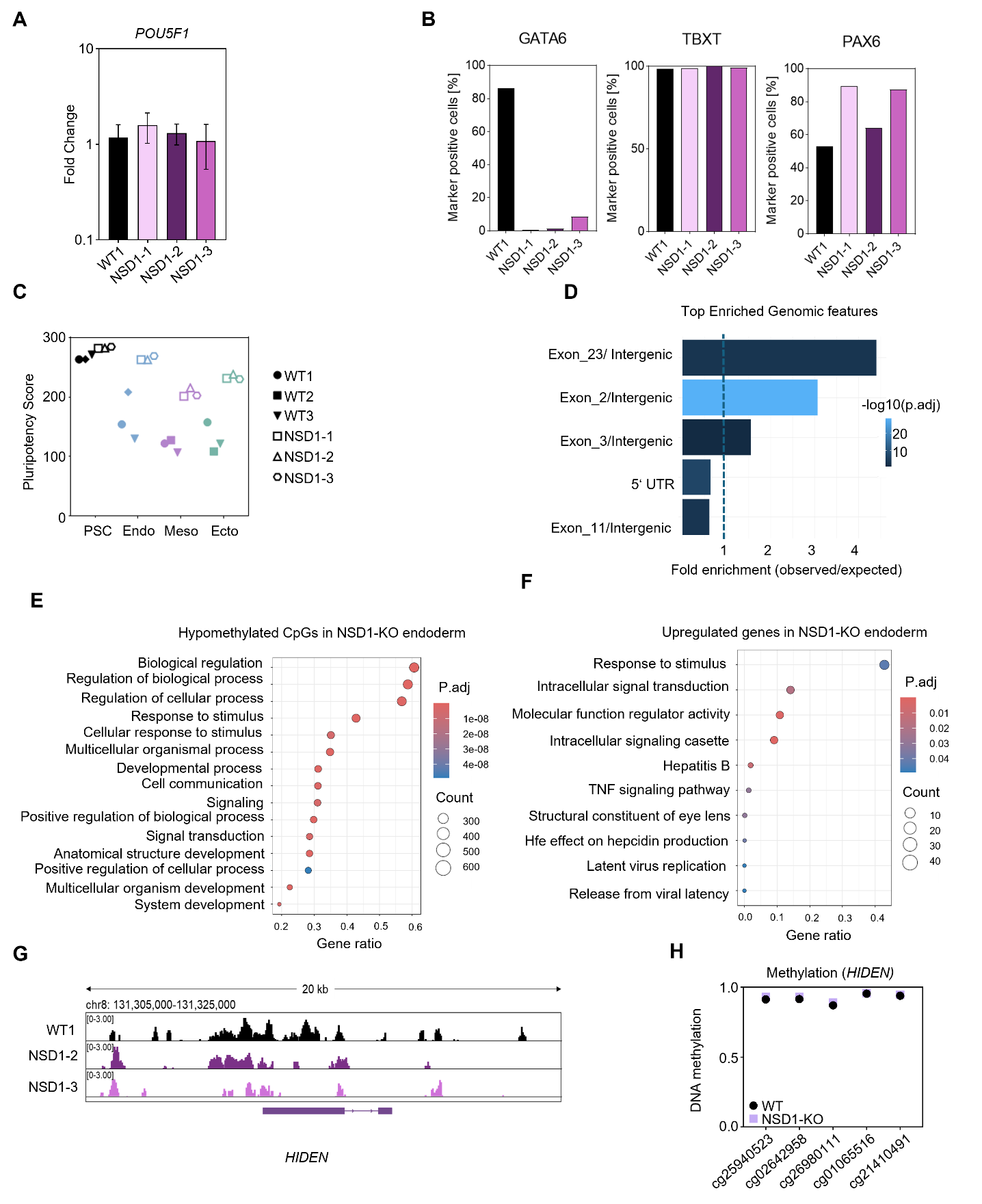


## Supplementary Figure 3: Differentiation defects in NSD1-KO

**(A)** Gene expression of *POU5F1* in undifferentiated NSD1-KO iPSCs. Mean fold changes were normalized to *GAPDH* and expression of *POU5F1* in WT iPSCs (± standard deviation). Statistical analysis was performed with an unpaired t-test. **(B)** Quantification of GATA6, TBXT and PAX6 immunofluorescence signal from WT and NSD1-KO cells (n=1 for each clone). 2 to 3 images containing 200-400 cells were measured and the percentage of cells positive for the respective marker is plotted. **(C)** Pluripotency Score of WT (n=3) and NSD1-KO (n=3) iPSCs in undifferentiated state and upon endodermal, mesodermal, and ectodermal differentiation. The Pluripotency Score was calculated using the sum of the beta-values of three pluripotency-specific CpG sites [37]. **(D)** Association of hypomethylated CpG sites in NSD1-KO endoderm with genomic regions. **(E)** Gene set enrichment analysis of hypomethylated CpG sites in NSD1-KO endoderm (n=3). **(F)** Gene set enrichment analysis of upregulated genes in NSD1-KO endoderm (n=3). **(G)** Visual representation of H3K36me2 enrichment at the *HIDEN* genomic region for WT and NSD1-KO iPSCs. **(H)** Beta-values of CpG sites close to lncRNA HIDEN in WT and NSD1-KO iPSCs (n=3).

## Supplementary Table 1: Antibodies used in this study

| **Primary antibodies** | | | | | | |
| --- | --- | --- | --- | --- | --- | --- |
| **Name** | **Host** | **Application** | **Dilution** | **Company** | **Clonality** | **Catalogue-Nr.** |
| Anti-GAPDH;  HRP-conjugated | Mouse | WB | 1:10,000 | Proteintech | monoclonal | HRP-60004 |
| Anti-H3K36me2 | Rabbit | WB | 1:2,500 | Abcam | polyclonal | ab9049 |
| Anti-H3K36me2 | Rabbit | ChIP | 4 μg | Abcam | polyclonal | ab9049 |
| Anti-H3K36me2 | Rabbit | IF | 1:2,000 | Abcam | polyclonal | ab9049 |
| Anti-OCT3/4 | Mouse | IF | 1:50 | Santa Cruz | monoclonal | sc-5279 |
| Anti-GATA6 | Rabbit | IF | 1:1,600 | Cell Signaling | monoclonal | 5851S |
| Anti-Brachyury | Goat | IF | 1:100 | R&D systems | polyclonal | AF2085 |
| Anti-PAX6 | Mouse | IF | 1:100 | Santa Cruz | monoclonal | sc-53108 |
| **Secondary antibodies** | | | | | | |
| **Name** | **Host** | **Target** | **Application** | **Dilution** | **Company** | **Catalogue-Nr.** |
| Alexa Fluor™ 594 | Goat | Rabbit | IF | 1:200 | Invitrogen | A-11012 |
| Alexa Fluor™ 594 | Goat | Mouse | IF | 1:200 | Invitrogen | A-11032 |
| Alexa Fluor™ 488 | Goat | Mouse | IF | 1:200 | Invitrogen | A-11029 |
| Alexa Fluor™ 594 | Donkey | Goat | IF | 1:200 | Invitrogen | A-11058 |
| Peroxidase AffiniPure™ | Goat | Rabbit | WB | 1:5,000 | Jackson ImmunoResearch | 111-035-003 |

## Supplementary Table 2: Primers used in this study

| **Taqman assays** | | |
| --- | --- | --- |
| **Target** | **Assay ID** |  |
| *POU5F1* | Hs04260367_gH |  |
| *GATA6* | Hs00232018_m1 |  |
| *T* | Hs00610080_m1 |  |
| *PAX6* | Hs01088114_m1 |  |
| *GAPDH* | Hs02758991_g1 |  |
| **qRT-PCR Primers** | | |
| **Name** | **Sequence** |  |
| *POU5F1* Fwd | GGGGGTTCTATTTGGGAAGGTA |  |
| *POU5F1* Rev | ACCCACTTCTGCAGCAAGGG |  |
| *GATA6* Fwd | CTCAGTTCCTACGCTTCGCAT |  |
| *GATA6* Rev | GTCGAGGTCAGTGAACAGCA |  |
| *FOXA2* Fwd | GCACTCGGCTTCCAGTATGCTG |  |
| *FOXA2* Rev | TCACGGAGGAGTAGCCCTCG |  |
| *GATA4* Fwd | GGCCTCTACATGAAGCTCCACG |  |
| *GATA4* Rev | CTGAAGGAGCTGCTGGTGTCTT |  |
| *TBXT* Fwd | CAGTGGCAGTCTCAGGTTAAGAAGGA |  |
| *TBXT* Rev | CGCTACTGCAGGTGTGAGCAA |  |
| *HIDEN* Fwd | TCACCGGTCCTCTTGTGTTG |  |
| *HIDEN* Rev | TTCTTTTCCAAAGCCGCTGA |  |
| *GAPDH* Fwd | GAAGTTGAAGGTCGGAGTC |  |
| *GAPDH* Rev | GAAGATGGTGATGGGATTTC |  |
| **Pyrosequencing primers** | | |
| **CpG site** | **Name** | **Sequence** |
| cg00661673 | cgSC1 Fwd | GGTTGGAGTGTATTGGTGTAA |
|  | cgSC1 Rev | Biotin-AATCCCAACCTTTATACATATTAATTCTT |
|  | cgSC1 Seq | GTTGAGATTATAGGTGTGA |
| cg00933813 | cgSC2 Fwd | AGGTTGGTTATGAATTTTTGGTTTTAAGTA |
|  | cgSC2 Rev | Biotin- ATACCCTACCTTCCTTTCATTTATATTC |
|  | cgSC2 Seq | TTGGGATTATAGGTGTG |
| cg21699252 | cgSC3 Fwd | GATGTTGAGGGTTAGGGGGTAATT |
|  | cgSC3 Rev | Biotin- CCTAAAACTCTAAAAATCTTTCTCCCTAAA |
|  | cgSC3 Seq | TGAAGGTTTTTTTAGTTTTGA |
| cg20548013 | cgE1 Fwd | GAATAGTATATGGTTGGTTGGGAAAGT |
|  | cgE1 Rev | Biotin- CCAAAAAAAAAAAATACCTTTACTATCACT |
|  | cgE1 Seq | AGGAGTTATTTTATTATATTGGAG |
| cg14521421 | cgE2 Fwd | GGGATGTTGTGGATGGTAAAA |
|  | cgE2 Rev | Biotin- ACTCCCACATCTAAACACCTAA |
|  | cgE2 Seq | AGGGGTGTGGGAAGT |
| cg08913523 | cgE3 Fwd | GGGAGAGGGATTTATTATTAGGT |
|  | cgE3 Rev | Biotin- ACCCCCTCCTTCAACTATAAT |
|  | cgE3 Seq | GGTTTGAGAAAGAAGTTAG |
| cg14708360 | cgM1 Fwd | AGGGTAAGGTTGTTTTGTTTAGTTTAT |
|  | cgM1 Rev | Biotin- TCATACCTTTAAAACCCACAACTAAAAT |
|  | cgM1 Seq | ATTAGGGTTTTGGTTTTATT |
| cg08826152 | cgM2 Fwd | TGAGTTTGGTTAGTTTAGTTATAGGT |
|  | cgM2 Rev | Biotin- CATCCCTAAAACAAACAAAAAACAATT |
|  | cgM2 Seq | ATTTGTTGTTGAGGTTTTTAATA |
| cg11599718 | cgM3 Fwd | ATGGTTTGGTATAGAAAGTTTATGG |
|  | cgM3 Rev | Biotin- ATACTTTCATCTCTTCTAATACCTTTAAC |
|  | cgM3 Seq | GTTTTGTGGGTGGGG |
| cg01907071 | cgEC1 Fwd | GGGGTTTTGAAAGTAAATGTGT |
|  | cgEC1 Rev | Biotin- TTCCAACTCACTAAAAAACACTTC |
|  | cgEC1 Seq | AGTAAATGTGTTGAAAGTT |
| cg18118164 | cgEC2 Fwd | AGTGGGAGTAAATGAGTTTAGT |
|  | cgEC2 Rev | Biotin- CAATTTCAAAATCTCCATCTCAAAATATCA |
|  | cgEC2 Seq | TTTTAGGGTAAGAAAATATAGATAG |
| cg13075942 | cgEC3 Fwd | GGGAGATTTTAGTTTTTTTTGTAGGG |
|  | cgEC3 Rev | Biotin- CCCAATATTATAATTCTTAACACCTCTCAT |
|  | cgEC3 Seq | AGTTTTTTTTGTAGGGATTTT |

## Supplementary Table 3: Endodermal and mesodermal regulator genes downregulated in NSD1-KO endodermal cells

| Gene | Function | log2FC |
| --- | --- | --- |
| *KLF5* | Endoderm regulator | -2.89682 |
| *FOSL2* | Endoderm regulator | -2.86778 |
| *HHEX* | Endodermal regulator | -8.65559 |
| *GATA6* | Endodermal regulator | -8.41851 |
| *EPHB3* | Endodermal regulator | -7.78796 |
| *FOXA1* | Endodermal regulator | -7.06227 |
| *LHX1* | Endodermal regulator | -6.89848 |
| *FOXP4* | Endodermal regulator | -6.79198 |
| *FLRT3* | Endodermal regulator | -6.72687 |
| *MYCT1* | Endodermal regulator | -6.67366 |
| *FOXA2* | Endodermal regulator | -6.1798 |
| *SMARCD3* | Endomesoderm regulator | -4.70637 |
| *BMP2* | Mesoderm regulator | -4.19813 |
| *FOXC1* | Mesoderm regulator | -4.14599 |
| *BMP7* | Mesoderm regulator | -2.40679 |
| *NOTCH1* | Mesoderm regulator | -1.12609 |
| *ANKRD1* | Mesoderm regulator | -5.56478 |
| *TWIST1* | Mesoderm regulator | -4.98244 |
